# Influence of Bile Acids in Hydrogel Pharmaceutical Formulations on Dissolution Rate and Permeation of Clindamycin Hydrochloride

**DOI:** 10.3390/gels8010035

**Published:** 2022-01-05

**Authors:** Nebojša Pavlović, Isidora Anastasija Bogićević, Dragana Zaklan, Maja Đanić, Svetlana Goločorbin-Kon, Hani Al-Salami, Momir Mikov

**Affiliations:** 1 Department of Pharmacy, Faculty of Medicine, University of Novi Sad, Hajduk Veljkova 3, 21000 Novi Sad, Serbia; isidora-anastasija.bogicevic@mf.uns.ac.rs (I.A.B.); dragana.zaklan@mf.uns.ac.rs (D.Z.); svetlana.golocorbin-kon@mf.uns.ac.rs (S.G.-K.); 2 Department of Pharmacology, Toxicology and Clinical Pharmacology, Faculty of Medicine, University of Novi Sad, Hajduk Veljkova 3, 21000 Novi Sad, Serbia; maja.djanic@mf.uns.ac.rs (M.Đ.); momir.mikov@mf.uns.ac.rs (M.M.); 3 Biotechnology and Drug Development Research Laboratory, Curtin Health Innovation Research Institute, School of Pharmacy and Biomedical Sciences, Curtin University, Bentley, WA 6102, Australia; hani.al-salami@curtin.edu.au

**Keywords:** topical drug, clindamycin, permeability, flux, gel

## Abstract

Clindamycin hydrochloride is a widely used antibiotic for topical use, but its main disadvantage is poor skin penetration. Therefore, new approaches in the development of clindamycin topical formulations are of great importance. We aimed to investigate the effects of the type of gelling agent (carbomer and sodium carmellose), and the type and concentration of bile acids as penetration enhancers (0.1% and 0.5% of cholic and deoxycholic acid), on clindamycin release rate and permeation in a cellulose membrane in vitro model. Eight clindamycin hydrogel formulations were prepared using a 2^3^ full factorial design, and they were evaluated for physical appearance, pH, drug content, drug release, and permeability parameters. Although formulations with carbomer as the gelling agent exerted optimal sensory properties, carmellose sodium hydrogels had significantly higher release rates and permeation of clindamycin hydrochloride. The bile acid enhancement factors were higher in carbomer gels, and cholic acid exerted more pronounced permeation-enhancing effects. Since the differences in the permeation parameters of hydrogels containing cholic acid in different concentrations were insignificant, its addition in a lower concentration is more favorable. The hydrogel containing carmellose sodium as a gelling agent and 0.1% cholic acid as a penetration enhancer can be considered as the formulation of choice.

## 1. Introduction

Topical antibiotics are currently used in a wide range of dermatological conditions, including acne, rosacea, impetigo, prevention of wound infections, among others [1]. The main advantage of topical therapy is the reduction or elimination of many systemic adverse effects. However, their use is restricted to less severe cases or superficial lesions, due to poor penetration to deeper skin layers, which significantly varies depending on the patient’s skin condition [2]. Topical medicines are usually intended to produce the pharmacological action at specific sites in the viable epidermis or upper dermis, and the ability of a drug molecule to penetrate the skin layers depends on its physicochemical properties and the characteristics of a carrier base [3].

Topical antibiotic medicines are available in many pharmaceutical dosage forms such as gels, creams, ointments, sprays, and liquids, containing an antibiotic drug alone or in combination with other drugs [4]. Semisolid dosage forms include a wide range of dosage forms possessing unique characteristics, and they are advantageous in terms of their easy application, rapid formulation, and ability to topically deliver various drug molecules [5]. Among them, hydrogels have a great significance for topical drug delivery due to their hydrophilic viscoelastic properties and the ability to absorb and retain large amounts of water, while maintaining their structure, mechanical strength, and elasticity [6]. Their specific properties such as moisture retention, exudate absorption, and gas permeability make them ideal as a vehicle for topical wound healing drug delivery [7].

Clindamycin, a lincosamide antibiotic, is widely used in therapy of mild to moderate cases of acne and skin infections. Clindamycin has been shown to be effective, safe, and well-tolerated after topical application, and it is currently available in a variety of different vehicles differing in the amount of water, alcohol and oil, including lotions, solutions, gels, and foams [8]. Although widely used in dermatology, the major issue of conventional clindamycin topical formulations for effective treatment is the hydrophilic nature of clindamycin (log P = 0.5), not suitable for skin penetration and accumulation in the pilosebaceous structures as lipophilic environment [9,10]. Clindamycin is a water-soluble weak base (pKa 7.72), which is mostly in ionized form at physiological pH [11], and it is usually classified in class III of the Biopharmaceutics Classification System (BCS), with good aqueous solubility and poor membrane permeability. Based on the drawbacks of clindamycin itself and its conventional formulations, several vesicular and particulate nanodelivery systems of clindamycin have been developed in order to enhance the delivery of clindamycin to the intended site of action after topical application [9,12,13,14,15].

Penetration enhancers have not been extensively studied in topical formulations of clindamycin. However, it was shown that significantly higher skin concentration of clindamycin phosphate in the viable skin layer was achieved when clindamycin phosphate gel was applied after the pretreatment of the skin with adapalene as a penetration enhancer [16]. To the best of our knowledge, topical formulations of clindamycin with bile acids have not been previously investigated.

The use of bile acids in both conventional dosage forms and novel micellar, vesicular, and polymer-based drug delivery systems has been increasingly investigated due to their biocompatibility and specific physicochemical properties [17]. Namely, bile acids may act as permeation enhancers not only by increasing the solubility of hydrophobic drugs, but also by increasing the fluidity of biological membranes, thus, improving the bioavailability of both hydrophilic and hydrophobic drugs [18]. In addition, the most common interaction of bile acids with drugs is ion-pairing, and the formed complexes may have either higher or lower polarity compared to the drug molecule itself. The pKa values of unconjugated bile acids are approximately five, and they exist in anionic form as bile salts at physiological conditions. Accordingly, they may directly interact with basic (cationic) drugs and thereby impact their aqueous/lipid transition. The formation of hydrophobic ionic complexes with bile salts is particularly significant for drugs belonging to the BCS class III, which possess high aqueous solubility and poor intestinal permeability [19].

Permeation-enhancing effects of bile acids were mostly studied following the peroral administration of drugs, although they can modulate the transport of drugs when administered via other routes as well. It was demonstrated that bile salts can enhance the penetration of drugs into the stratum corneum of skin, by interacting with keratin filaments and hemidesmosomes, leading to corneocyte disruption and the increase in paracellular drug transport [20]. Since the mechanism of action of drug penetration enhancers, in general, involves intensive interaction with the lipids or the corneocytes of the stratum corneum, they have a high potential to cause skin irritation [21]. Hydrophobicity is the most important determinant of bile acid toxicity, and hydrophobic bile acids such as deoxycholic acid have been reported to exert more toxic effects following systemic and local application [22]. Therefore, low physiologically safe bile acid concentrations were used in hydrogel formulations in this study.

Considering all the positive characteristics of hydrogels, the biopharmaceutical properties of clindamycin, and the potential of bile acids to be used as drug release modifiers and drug penetration enhancers, the aim of this study was to formulate 1% clindamycin hydrogel formulations for topical application with good physical properties and adequate release of the active substance. Furthermore, the objective was to investigate the effects of the type of gelling agent, as well as the type and concentration of bile acids as penetration enhancers, on the clindamycin dissolution rate and its permeation in a cellulose membrane in vitro model.

## 2. Results and Discussion

### 2.1. Physical Appearance

The acceptance of pharmaceutical semisolid dosage forms and cosmetic products by patients and consumers depend not only on the efficacy of the active substances but also on the aesthetic characteristics of the product, i.e., physical appearance and sensory properties [23]. The physical parameters and pH values of the hydrogels formulated in this study are summarized in Table 1. Quantitative composition of the tested hydrogel formulations is shown in the ‘Materials and Methods’ section.

The control hydrogel formulation with carbomer as gelling agent (C1) was colorless, transparent, with excellent homogeneity and soft consistency, suitable for dermal application. On the other hand, the control gel with carmellose sodium as a gelling agent (C2) was yellowish with very viscous consistency, and the individual lumps of gelling agent could be observed. The addition of bile acids to G1–G8 formulations did not have a significant effect on the consistency, i.e., it remained approximately the same as in the corresponding control hydrogels. However, the addition of deoxycholic acid significantly impaired the homogeneity of formulations G1, G2, G5, and G6, especially at a higher concentration of 0.5%. The homogeneity of hydrogels with cholic acid as penetration enhancer was approximately the same as in the corresponding control formulations.

Formulations of clindamycin carbomer hydrogels with added bile acids (G1–G4) had pH values in a range from 6.5 to 6.7, and they were slightly lower in comparison to the pH of C1 control formulation (pH 6.8). Likewise, clindamycin hydrogels with carmellose sodium and bile acids (G5–G8) had pH values in a range from 6.8 to 7.0, slightly reduced when compared to the pH of C2 control formulation (pH 7.1). When comparing the acidity of carbomer and carmellose sodium hydrogels in general, lower pH values were shown for hydrogels with carbomer.

The pH values of prepared hydrogel formulations were in the range that is considered acceptable to avoid the risk of irritation upon application to the skin [24]. The physiological pH of the stratum corneum is 4.1–5.8 and inflammatory skin diseases, including rosacea and acne, exhibit a disturbed skin barrier with increased pH [25]. In these cases, the topical application of slightly acidic preparations that may establish physiological microbiota and repair skin barrier is recommended. On the other hand, considering the alkaline nature of clindamycine [11], lower pH values increase the ionization of this drug substance and decrease its permeability.

### 2.2. Clindamycin Content in Hydrogel Formulations

The results of the drug content determination in hydrogel formulations are shown in Table 2. The content of clindamycin hydrochloride differed minimally among tested formulations, and it met the expected value of 1.13%, which corresponds to the content of 1% clindamycin base. The percentage of clindamycin hydrochloride in the gel formulations ranged from 98.42% to 99.96% of the desired drug content, which indicates that the hydrogel formulations were made by an adequate method of preparation. In addition, the low values of standard deviations indicate the high homogeneity of clindamycin hydrochloride in hydrogels. However, clindamycin is a suitable drug substance for hydrophilic pharmaceutical formulations considering its high aqueous solubility and stability in a wide pH and temperature range [26].

### 2.3. Dissolution Profiles and Permeation Parameters of Clindamycin in Hydrogel Formulations

**Figure 1:**
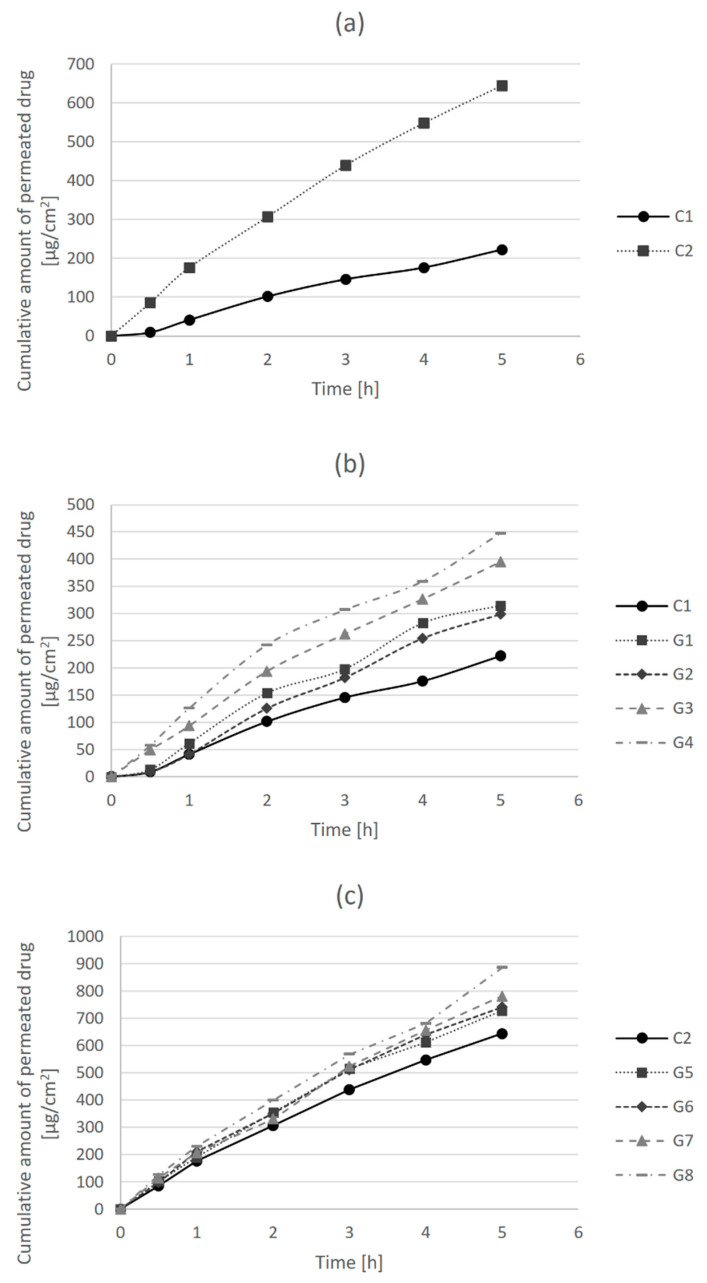
Dissolution profiles of clindamycin hydrochloride from hydrogel formulations: (**a**) control formulations C1 and C2; (**b**) hydrogels G1-G4 in comparison to control gel C1; and (**c**) hydrogels G5–G8 in comparison to control gel C2.

Drug permeability is defined as the ability of a drug to pass across a biological membrane, and it is particularly important from the aspect of drug action, since the drug substance needs to reach the desired site of action in order to exert its effects [27]. Permeability parameters of clindamycin hydrochloride in tested hydrogel formulations were determined using in vitro drug release and permeation test across an artificial cellulose membrane. The amount of clindamycin hydrochloride permeated per unit surface area (µg/cm^2^) was plotted versus time, and the obtained dissolution profiles are presented in Figure 1.

In Figure 1a, it can be observed that the release of clindamycin and its permeation across the membrane over time was higher in the control hydrogel formulation with carmellose sodium (C2) than in the carbomer control gel (C1). Figure 1b shows the differences in the release and permeation of clindamycin in carbomer hydrogel formulations (C1 and G1–G4). The permeability of clindamycin increased with the addition of bile acids, particularly after the addition of cholic acid in a higher concentration (G4). Similarly, the permeability of clindamycin in carmellose sodium gels increased with the addition of bile acids (Figure 1c). It was more pronounced after the addition of cholic acid (G7, G8) than deoxycholic acid (G5, G6), and slightly more after the addition of cholic acid at a higher concentration (G8) in comparison to a lower concentration (G7).

The values of clindamycin permeability parameters in different hydrogel formulations, such as steady-state flux (J_ss_), the permeability coefficient (Pm) and the permeation enhancement factor (EF) were calculated from dissolution curves, and they are presented in Table 3. Steady-state flux (J_ss_) is a parameter that shows the amount of clindamycin that permeates one cm^2^ of membrane per hour. Permeation enhancement factor (EF) is a parameter that demonstrates the effectiveness of penetration enhancers by comparing the permeation rate of clindamycin in the presence and absence of bile acids as enhancers.

In the case of carbomer hydrogel formulations (G1–G4), the largest increase in flux of 1.74 times compared to the flux of C1 control formulation was shown for the G4 formulation with cholic acid in a concentration of 0.5%. Likewise, the G8 formulation of carmellose sodium gel with 0.5% cholic acid had the highest EF value among carmellose sodium hydrogel formulations (G5–G8) in comparison to the C2 control formulation. Although the enhancement factors of bile acids were higher in the carbomer gels in comparison to the carmellose sodium gels, when comparing the flux of carmellose sodium hydrogels (G5–G8) to the flux of carbomer control gel C1, the enhancement factors were in the range from 3.06 (G5) to 3.65 (G8). This is very important since the formulation of 1% clindamycin in carbomer hydrogel (C1) is the most often used for acne treatment in practice. The enhancement of drug release and permeation are not the consequence of the presence of bile acids as permeation enhancers only but also of the choice of gelling agent, i.e., carmellose sodium instead of carbomer.

Penetration enhancers are usually surfactants that have the potential to alter the lipid integrity within the stratum corneum, and their choice in formulation development is based on drug efficacy and their own effects on the skin as well [28]. Bile acids have been extensively investigated for their drug transport enhancement properties across different biological membranes, following the specific routes of drug administration [19].

The influence of bile acid co-administration on skin permeation of drugs has also been studied. It was demonstrated that the addition of sodium glycocholate significantly improved the permeation of the tromethamine salt of non-steroidal anti-inflammatory drug (NSAID) ketorolac across rat skin [29]. Furthermore, bile salts sodium tauroglycocholate and sodium deoxycholate were shown to enhance in vitro permeation of theophylline through shed snake skin, and their permeation-enhancing activity was not concentration-dependent [30]. The hydrogel formulation containing corticosteroid drug betamethasone-17-valerate and sodium deoxycholate as penetration enhancer was tested for its in vitro skin permeation characteristics and in vivo anti-inflammatory activity. It was demonstrated that transdermal permeation of this corticosteroid across the rat skin was eight-fold elevated in comparison to the commercial cream with the same concentration of the active ingredient. Additionally, in vivo anti-inflammatory activity was positively correlated with in vitro drug permeation [31].

Our results were in accordance with the above-mentioned studies, and it is assumed that one of the potential mechanisms by which bile acids increase clindamycin permeability is the formation of hydrophobic ionic complexes, given that bile acids as weak acids exist in anionic form in aqueous solutions and clindamycin as a weak base is in cationic form.

### 2.4. Molecular Mechanics Calculations of Clindamycin-Bile Acids Interactions

**Figure 2:**
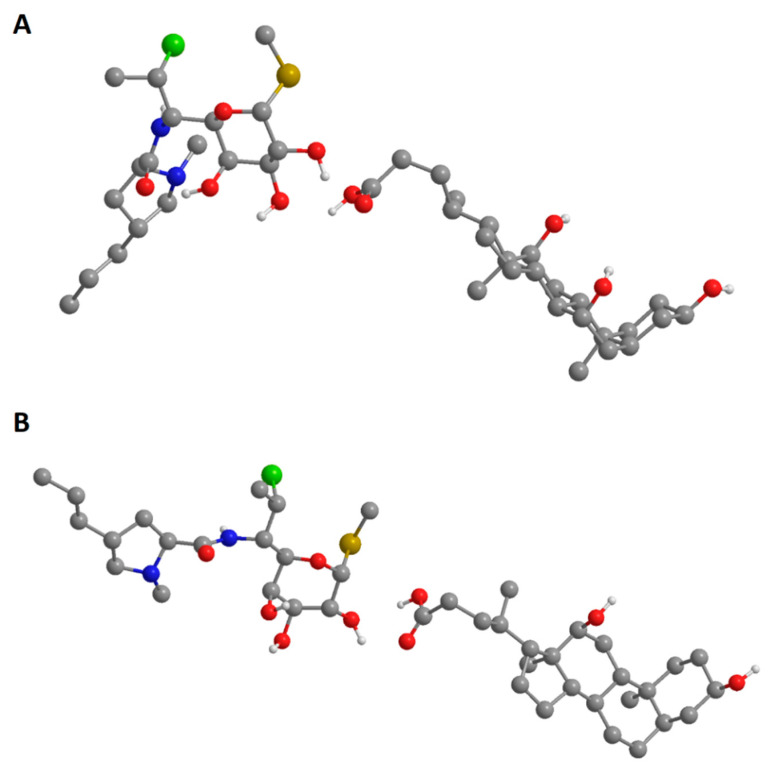
Geometrically optimized three-dimensional structures of clindamycin/cholic acid (**A**) and clindamycin/deoxycholic acid (**B**) complexes.

The interactions of clindamycin hydrochloride and bile acids were investigated also by molecular mechanics calculations (MM2), using the geometrically optimized 3D structures. The total energies, as the sum of stretching, bending, torsion, electrostatic, and non-bonded interaction energies, were calculated. The minimized total energies of clindamycin hydrochloride, cholic acid, and deoxycholic acid were 29.3047 kcal/mol, 52.1559 kcal/mol, and 48.8076 kcal/mol, respectively. The total energies of both clindamycin/deoxycholic acid and clindamycin/cholic acid complexes were lower than the sum of the potential energies of the two single components optimized by molecular mechanics calculations, indicating that the formation of the complexes induced a stabilization of the system (Table 4). Clindamycin/cholic acid complex was shown to be more stable in comparison to the clindamycin/deoxycholic acid complex. From the calculation of the partial energy distribution it can be concluded that the non-1,4 Van der Waals (non-1,4-VDW) energies and dipole–dipole attractive forces between the polar ends of molecules had a major stabilizing effect, particularly in the case of clindamycin/cholic acid complex (Figure 2).

The formation of hydrophobic ionic complexes has been demonstrated for deoxycholic and ursodeoxycholic acid with polar antibiotic drugs kanamycin, amikacin, and vancomycin, which are characterized by low penetration into the cells. The obtained complexes were structurally characterized, and they showed higher inhibition of *Staphylococcus aureus* growth in comparison to the parent drugs. The aminoglycoside complexes, in particular kanamycin-deoxycholic acid, exhibited strong inhibition of biofilm formation, since the aminoglycosides kanamycin and amikacin are strongly basic compounds that exist as polycations at physiological pH [32]. The formation of hydrophobic ion complexes with bile salts has been suggested also for trospium chloride, a polar quaternary amine with low membrane permeability. Both structure and concentration of bile salts influenced the ion-pairing and the transport of this cationic drug across Caco-2 cells and excised rat jejunum [33].

Most studies investigating the drug permeation enhancing effects of various bile acids have confirmed their potential to be used as permeation enhancers in pharmaceutical formulations, and sodium deoxycholate has been shown to be the most effective permeation enhancer in general. The effect of cholic and deoxycholic acid on membrane transport differs due to differences in their chemical structures, such as the number of hydroxyl groups, which affects their hydrophobicity. In contrast to the previously analyzed studies [30,31], cholic acid in our study proved to be a more effective permeation enhancer of clindamycin hydrochloride than deoxycholic acid. This can be explained by the formation of more stable hydrophobic complex of cholic acid with clindamycin in comparison to deoxycholic acid, as demonstrated by molecular mechanics calculations. In addition, it can be assumed that the lower values of deoxycholic acid permeation-enhancing properties are a consequence of its crystallization in the hydrophilic gel structure due to its low aqueous solubility. This issue could be solved by the formation of sodium salts of deoxycholic acid, but it would also lead to an increase in the formulation pH, which would not be adequate for application to the site of infection and inflammation [25]. The other possibility is to increase the share of propylene glycol as a co-solvent in the formulations, which would not affect the pH of hydrogels.

## 3. Conclusions

While the formulations with carbomer as a gelling agent exerted optimal sensory properties, carmellose sodium hydrogels had significantly higher release rates and permeation of clindamycin hydrochloride. The type and concentration of bile acid affected the dissolution rate and permeation of clindamycin. The bile acid enhancement factors were higher in the carbomer gels in comparison to the carmellose sodium gels. In general, the best dissolution profiles, i.e., the highest values of permeation parameters, were shown for formulations with 0.5% cholic acid. Since the differences in the permeation parameter values of hydrogel formulations containing cholic acid in concentrations of 0.1% and 0.5% were insignificant, the addition of cholic acid as a permeation enhancer in a lower concentration is more favorable.

Based on all results, it can be concluded that, out of eight tested clindamycin hydrogel formulations, the formulation G7 containing carmellose sodium as a gelling agent and 0.1% cholic acid as a permeation enhancer is the formulation of choice. The obtained results represent a good basis for further in vivo studies of the tolerability and efficacy of the optimal clindamycin hydrogel formulation in various dermatological diseases.

## 4. Materials and Methods

### 4.1. Materials

The following materials were used in this study: clindamycin hydrochloride (Fagron, Terrassa, Spain), deoxycholic acid and cholic acid (Sigma-Aldrich, Steinheim, Germany), carmellose sodium (Carl Roth, Karlsruhe, Germany), carbomer 940 (Carbopol^®^ 940, Lubrizol Advanced Materials, Cleveland, OH, USA), and triethanolamine (TEA) and propylene glycol (Lach:ner, Brno, Czechia). All chemicals used were of analytical grade (*p.a.*) and were used without any further chemical modification.

### 4.2. Hydrogel Formulation Design and Preparation Method

Eight formulations of 1% clindamycin hydrochloride gel were prepared with two different gelling agents, two different bile acids as permeation enhancers in two different concentrations, adapting a 2^3^ full factorial design. Carbomer 940 (*Carbomera*, Ph. Eur.) and sodium carboxymethyl cellulose (*Carmellosum natricum*, Ph. Eur.) were used as gelling agents (independent variable *X*_1_). The release and permeation enhancers in the hydrogel formulations were deoxycholic acid and cholic acid (independent variable *X*_2_) in concentrations of 0.1% and 0.5% (independent variable *X*_3_). Factors and levels for 2^3^ factorial design are presented in Table 5 and the quantitative composition of the prepared formulations is shown in Table 6.

All hydrogel formulations were extemporaneously prepared using standard utensils and equipment. The control hydrogels C1 and C2 were compounded according to the preparation procedures for ‘Carbomeri mucilago’ and ‘Carmellosi natrici mucilago’, respectively, as described in the current national formulary for extemporaneous formulations (Magistral formulae 2008 of the Republic of Serbia) [34], where clindamycin hydrochloride was first dissolved in water. In the C1 formulation, carbomer 940 was first dispersed in clindamycin aqueous solution and left to swell and hydrate for 1 h, i.e., to form a homogeneous dispersion without lumps. Afterwards, 10% TEA aqueous solution was added in order to neutralize the carbomer (polyacrylic acid), as well as propylene glycol as humectant. The resulting gel was stirred well and allowed to swell completely. In the C2 control gel, carmellose sodium was dispersed in an aqueous solution of clindamycin by adding this solution in portions to an enamel dish with a gelling agent and mixing with a pestle until a homogeneous dispersion was formed. After the carmellose sodium was fully hydrated, propylene glycol was added and mixed well. G1–G4 hydrogel formulations were prepared according to the procedure described for control gel C1, and G5–G8 gel formulations according to the procedure for C2 gel, with bile acids being added in formulations in the form of propylene glycol solutions.

### 4.3. Visual Examination and Measurement of pH

Following the preparation, hydrogel formulations were set in a glass container and inspected visually for their color, homogeneity, and consistency. For the homogeneity testing by visual inspection, hydrogels were set between two glass plates in order to observe the potential presence of any lumps and aggregates.

The pH of hydrogel formulations was determined by using a digital pH meter (WTW inoLab, Weilheim, Germany), which was calibrated before each use with standard buffer solutions. The measurement of pH of each formulation was performed in triplicate, and average values were calculated [35].

### 4.4. Drug Content Determination

The content of clindamycin hydrochloride in hydrogel formulations was determined spectrophotometrically [36]. Samples (1 g) of hydrogel formulations were first dissolved in 100 mL of 0.05 M phosphate buffer pH 6.8 using ultrasound for 5 min. Phosphate buffer was freshly prepared according to the procedure of the US Pharmacopeia [37]. The solutions were further diluted 10-fold with the same solvent, and the absorbance was recorded at 210 nm against the corresponding phosphate buffer pH 6.8 as a blank. The content of clindamycin in each formulation was calculated using the external standard. The calibration curve of clindamycin hydrochloride in 0.05 M phosphate buffer pH 6.8 was linear in the concentration range of 1.25–20 µg/mL (y = 0.0066x + 0.0168, R^2^ = 0.998). The analysis was performed in triplicate.

### 4.5. Determination of In Vitro Release and Permeation of Clindamycin Hydrochloride

**Figure 3:**
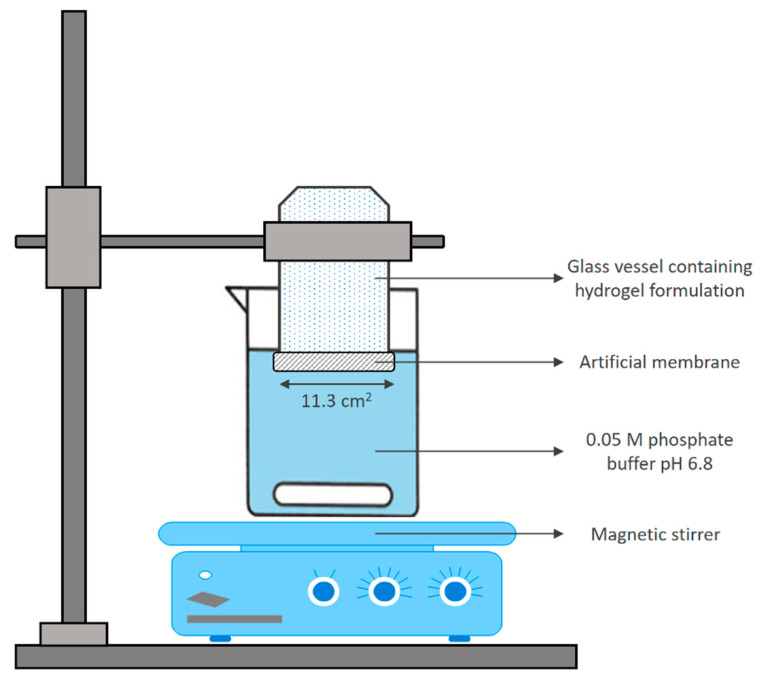
Schematic representation of in vitro drug release and permeation model.

An in vitro release and permeation test of clindamycin hydrochloride from hydrogel formulations was performed in an apparatus, resembling a Franz diffusion cell (Figure 3) [12]. Briefly, a regenerated cellulose dialysis membrane (Nadir^®^, mean pore size 15,000 Da) was stretched over the open end (surface 11.3 cm^2^) of a glass vessel containing the gel and made water tight using a rubber band. The vessel was immersed vertically in a beaker containing 300 mL of 0.05 M phosphate buffer pH 6.8 placed on a magnetic stirrer (Boeco MSH 140, Hamburg, Germany), set at 50 rpm. The test was performed at a room temperature of 25 °C. Phosphate buffer pH 6.8 was used as a dissolution medium, considering the good solubility of clindamycin hydrochloride in this medium, thus, eliminating the effect of intrinsic solubility on the dissolution profile and analyzing only the impact of pharmaceutical formulations on drug release and permeation. At predetermined time intervals for up to 300 min, 2 mL aliquots of the dissolution medium were withdrawn for analysis and were replaced with an equal volume of medium to maintain a constant volume. The samples were filtered, and the absorbance was measured spectrophotometrically at λ_max_ of 210 nm. The results were expressed as the mean values of three independent release experiments.

The amount of the drug permeated per unit surface area (µg/cm^2^) was plotted versus time (hours). From the dissolution curves, the steady-state flux J_ss_ (µg/cm^2^/h) was calculated for each formulation as the slope of the linear part of the curve (from 60th to 300th minute).

The permeability coefficient (P_m_) was then calculated according to the following equation [38].

P_m_ = J_ss_/C_d_;

where P_m_ is the permeability coefficient (cm/h), J_ss_ is the steady-state flux, and C_d_ is the concentration of the drug in the donor side, i.e., in gel formulations [39].

The enhancement factor (EF) was calculated as the ratio of the steady-state flux of the formulation containing the permeation enhancer and the steady-state flux of the corresponding control formulation without the permeation enhancer.

Negative and positive controls were performed before the analysis of clindamycin hydrogel formulations. The blank formulations (without clindamycin, but with and without bile acids) were first tested, and no interferences were identified at 210 nm. In addition, the suitability of this method has been confirmed using low permeable and highly permeable drugs.

### 4.6. Structural Modelling and Geometry Optimization

Initial 3D structures of clindamycin hydrochloride and bile acids (cholic acid and deoxycholic acid) were prepared using the Chem3D Ultra v. 16.0.1.4 program package. The initial 3D structure of clindamycin–bile acid complexes were constructed in the same way. Molecular geometries were optimized using MM2 force field calculations, as implemented in Chem3D Ultra software [40].

### 4.7. Statistical Analysis

All data were presented as a mean ± standard deviation (SD). For multiple group comparisons, one-way analysis of variance (ANOVA) followed by Tukey’s post hoc test at *p* < 0.05 was applied using SPSS software (version 23.0, IBM Institute Inc., Chicago, IL, USA).

## Figures and Tables

**Table 1:** Physical appearance and pH values of formulated hydrogels.

Formulation	Color	Homogeneity *	Consistency °	pH
C1	Transparent	5	3	6.8 ± 0.1
C2	Yellowish	4	5	7.1 ± 0.1
G1	Hazy white	3	3	6.6 ± 0.1
G2	Hazy white	2	3	6.6 ± 0.2
G3	Transparent	5	3	6.7 ± 0.2
G4	Transparent	5	3	6.5 ± 0.1
G5	Hazy yellowish	3	5	6.9 ± 0.0
G6	Hazy yellowish	2	5	7.0 ± 0.1
G7	Yellowish	4	5	6.9 ± 0.2
G8	Yellowish	4	5	6.8 ± 0.0

* Homogeneity: from 1 (nonhomogeneous) to 5 (excellent homogeneity); ° Consistency: from 1 (very low viscosity) to 5 (very high viscosity).

**Table 2:** Clindamycin content in hydrogel formulations.

Formulation	Clindamycin Content [%, g/100 g]	Relative Content of Clindamycin [%]
C1	1.12 ± 0.01	98.75 ± 1.27
C2	1.13 ± 0.02	99.62 ± 1.34
G1	1.11 ± 0.00	98.62 ± 0.07
G2	1.13 ± 0.01	99.56 ± 0.60
G3	1.13 ± 0.01	99.96 ± 0.47
G4	1.11 ± 0.00	98.42 ± 0.40
G5	1.13 ± 0.02	99.96 ± 1.54
G6	1.11 ± 0.01	98.48 ± 1.14
G7	1.12 ± 0.01	98.75 ± 0.74
G8	1.12 ± 0.02	98.89 ± 1.81

**Table 3:** Permeability parameters of clindamycin in hydrogel formulations.

Formulation	Linear Regression Equation	Coefficient of Determination, R^2^	Steady-State Flux, J_ss_ [µg/cm^2^/h]	Permeability Coefficient, P_m_ [cm/h × 10^3^]	Enhancement Factor (EF)
C1	y = 43.64x + 6.2349	0.9874	43.64	3.862	1
C2	y = 117.86x + 69.187	0.9953	117.86	10.430	1
G1	y = 63.56x + 11.263	0.9774	63.56	5.625	1.46 *
G2	y = 64.36x − 12.872	0.9903	64.36	5.695	1.47 *
G3	y = 73.61x + 33.387	0.9914	73.61	6.514	1.69 *
G4	y = 76.03x + 67.981	0.9810	76.03	6.728	1.74 *
G5	y = 133.55x + 79.646	0.9878	133.55	11.819	1.13 **
G6	y = 135.16x + 85.278	0.9936	135.16	11.961	1.15 **
G7	y = 147.63x + 56.718	0.9940	147.63	13.065	1.25 **
G8	y = 159.29x + 74.819	0.9942	159.29	14.096	1.35 **

* vs. C1; ** vs. C2.

**Table 4:** The minimized total energies of clindamycin hydrochloride (E_C_), bile acids (E_BA_) and clindamycin-bile acid complexes (E_C+BA_).

Bile Acid	E_C_ (kcal/mol)	E_C_ + E_BA_ (kcal/mol)	E_C+BA_ (kcal/mol)	ΔE (kcal/mol)
Cholic acid	29.3047	81.4606	58.6516	−22.8090
Deoxycholic acid	29.3047	78.1123	66.6174	−11.4949

**Table 5:** Factors and levels for 2^3^ factorial design.

Independent Variable	Levels	
	−1	+1
*X*_1_: Type of gelling agent	Carbomer 940	Carmellose sodium
*X*_2_: Type of permeation enhancer	Deoxycholic acid	Cholic acid
*X*_3_: Concentration of permeation enhancer	0.1%	0.5%

**Table 6:** Quantitative composition of hydrogel formulations (% *wt*/*wt*).

Component	Hydrogel Formulation									
	G1	G2	G3	G4	G5	G6	G7	G8	C1	C2
Clindamycin HCl	1.13	1.13	1.13	1.13	1.13	1.13	1.13	1.13	1.13	1.13
Carbomer 940	0.5	0.5	0.5	0.5	-	-	-	-	0.5	-
Carmellose sodium	-	-	-	-	5	5	5	5	-	5
Deoxycholic acid	0.1	0.5	-	-	0.1	0.5	-	-	-	-
Cholic acid	-	-	0.1	0.5	-	-	0.1	0.5	-	-
TEA solution 10%	7	7	7	7	-	-	-	-	7	-
Propylene glycol	10	10	10	10	10	10	10	10	10	10
Purified water	100	100	100	100	100	100	100	100	100	100

## Data Availability

The data presented in this study are available in the article.
